# Improvisational Dance-Based Psychological Training of College Students’ Dance Improvement

**DOI:** 10.3389/fpsyg.2021.663223

**Published:** 2021-05-26

**Authors:** Xinyu Dou, Lin Jia, Jinchuan Ge

**Affiliations:** ^1^Conservatory of Music, Zhengzhou University, Zhengzhou, China; ^2^Faculty of the Professions, University of Adelaide, Adelaide, SA, Australia

**Keywords:** choreography, innovation, psychological disorders, semi-structured interview, AHP analytic hierarchy process

## Abstract

Dance creation involves complex psychological activities. Although previous studies have conducted extensive investigations on the psychological aspects of choreographers’ creations, little is known regarding the psychological barrier of choreographers in terms of creativity. The study aims to explore the psychological barrier of innovation in dance choreography, which is a kind of situation between mental illness and mental problems. The research shows that improvisational dance is a free dance with the human body as a material carrier, and it is a dance form that the dancer’s thinking is transformed into dance action to express their inner thoughts. The improvement of the potential creative thinking of dance creators through the improvisational dance movement and structure are explored. Meanwhile, the theoretical ideas of psychologists have been applied to the cultivation and improvement of improvisational dance creativity, which has made full certification and supplement. Moreover, through psychological experiments, it is proved that improvisational dance is an important way to release and develop creativity. Based on the theory of psychology, feasible suggestions are given to improve students’ creative ability in the process of improvisational dance teaching.

## Introduction

Dance is an attractive and enjoyable art form of creative expression. People have discovered this type of art for a long time, which has evolved with the progress of human society (H’Doubler, 1957). The proficiency of dance skills is usually assessed based on the performer’s physical skills, physical coordination, flexibility, and strength, as well as other subjective performances and aesthetic elements (Bläsing et al., 2012). As an important creator of dance art, the choreographer is responsible for designing and depicting the artistic practice of dance in terms of movement sequence and form content. Dancers vary their movements according to space, speed, rhythm, etc., and express them precisely and improve them according to the choreographer’s demonstration. In addition, the dancers perfect the movements according to the choreographer’s aesthetic and expressive abilities and dance style. In the current society full of commercial atmosphere, mass culture profoundly affects the aesthetic interest of young people and gives birth to a new generation of dance choreographers (Henfield et al., 2010). These new generation choreographers need to take into fusing the traditional techniques to create new trends in modern society, which is based on the current time and changes with the times, thus meeting and leading the audience’s continuous development of aesthetic interest (Long, 2006).

Dance creation involves complex psychological activities: dancers need to combine physical movements with cognitive skills to observe, execute, and coordinate complex movement patterns; however, the choreographer is faced with more activities about psychology from the creation period. Although the relationship between creativity and psychology, the mood of artists (such as architects, painters, and musicians) has been extensively studied, and the two are considered to have a significant correlation (Lauronen et al., 2004; Fink and Woschnjak, 2011; Gillam, 2013). Improvisational dance is the soul and beginning of modern dance, and its creation comes from external stimulation, which produces the expression of emotion to the art and expresses it from the inside to outside according to the experience and skills accumulated (Fisher et al., 2020). Improvisational dance is the ability each dancer needs to possess, and it is an emotional expression of the impulse that arouses instinct in a moment. This editing and creation technique is the most challenging, expressive, personalized, and the most real emotional externalization, which is a performing art that integrates body and mind; therefore, “improvisational dance is the source of all techniques of creation,” and is the most primitive form of dance origin.

Considering that excellent works with dance emerge in an endless stream in today’s society but cannot see the high peaks only in the plateau, it can be called the classic and opening work of the best. Freud’s psychoanalytic unconscious creativity theory and other related creativity theories are used to explore the important value of improvisation in dance training and the relationship between improvisational dance and creativity (De Oliveira Moreira and Drawin, 2015). On the one hand, the relevant psychological theories about improvisational dance in dance psychology are enriched. On the other hand, through the experimental research of scientific psychology, it is concluded that improvisational dance can be used as a training mode to open the body creativity, which not only can solve the body initiative, but also stimulate the body potential of the dancer. A positive and meaningful attempt on the application of creativity theory in the teaching of improvisational dance, is made. It is believed that it can make a supplement to the less relevant literature in China, provide a reference for this kind of data, and promote the improvement of the teaching method of improvisational dance. In this way, the practical experience in the teaching of improvisational dance can be upgraded to a theory with universal guiding significance, which can train original talents for dance education and provide a theoretical reference.

## Literature Review

### Research on Choreography

By searching the keyword of “choreography” in the CNKI, WanFang, CQVIP, and Web of Science database, it can be seen that due to the important role of choreography in the creation and performance of dance, a large number of existing literatures have been studied. Furthermore, most of the current studies are focus on the process of choreography. For example, Kirsh et al. (2000) conducted a detailed ethnographic study of the process of dance creation of a famous choreographer and his team. By recording interactive videos of choreographers and conducting extensive interviews with dancers as well as other observations and tests, it was found that choreographers used three main methods to improve creative quality and creativity: presentation, making-on, and tasking. Abecassis-Moedas and Gilson (2017) took 20 dance choreographers as samples, adopted qualitative research methods to discuss creative leadership in the creation of dance, and depend on the pattern coding to find that the driving forces of creative leadership in dance choreographers include perspective, project, and preference. Triana investigated the relationship between the creative thinking ability of choreographers and the quality of dance creation and pointed out that there was a positive correlation between the two (Triana, 2015). In addition, a large number of studies have discussed the creation process of dance choreographers (Nahrstedt et al., 2008; Felice et al., 2016), or analyzed and reviewed the creation of thoughts and the creation of dance styles of a famous choreographer (Powell, 1995; Giordano, 2000; Megill, 2018).

### Creativity and Innovation Ability

In the field of artistic creativity, highly creative people (such as artists or dancers) tend to be introverted, relatively high in emotion or neuroticism, more open to experience sharing and have a strong desire for achievement or performance (Haller and Courvoisier, 2010). For choreographers, body movements need to be designed using internal and external cues, and expressed in highly creative ways, from the most abstract ideas to the concrete, audience-aware forms of expression. Artists’ creativity is often thought of as an “internal process” that takes place only in the mind, and Kirsh (2014) offers a new and more interactive view that collaboration with objects, media, and other external objects, is also an essential part of creative thinking. In addition, Ludwig believes that the creativity of various art forms including the creation of dance includes three dimensions: the creator himself, the creating process, and the product of creativity (Ludwig, 1989). The research on the creative ability of artistic creation also includes its relationship with personality type and mental illness (Kaufman, 2005).

### Improvisational Dance and Creativity

Through sci-hub, JSTOR, Library Genesis, and other network data resources, as well as related works, improvisational dance, dance and, creativity are used as keywords to search the literature in recent years. In these research areas, the following aspects are summarized. By observing the improvisational dance performance, Łucznik (2015) realized how the dancers cooperate and create, and put forward some opinions. It was believed that dancers were not only the internal process of a single creator, but also the interaction among a group of improvised dancers from the mind, body, and environment. For group creativity, an improvisation score was used to test and video recording was used to stimulate the recall method. After scoring, the dancers got the score and reflected on their own process through video playback. The interdependence among the original knowledge strategies was discussed, such as imagery and the sense of feeling, the process of the group, the role of others in the process of creation, and the interaction between the body and the environment (Łucznik, 2015). Chung discussed that the dance symbol system was generally considered as a tool of expression rather than a creative tool; as an uncertain method, improvisational dance was regarded as a creative way to assist the body’s movement and development. Based on the theory of Laban Movement analysis, the motion symbol system was constructed. Moreover, an in-depth interview was conducted, which provided a comprehensive insight into the perspective of choreography (Chung and Hou, 2018). Fink and Woschnjak (2011) described how three different groups of professional dancers (ballet, modern, and jazz) differ greatly in the creative needs involved in their respective dance styles. In this regard, the author compared creativity, general psychological ability, and personality. The author’s research results show that modern dancers perform freely on stage and show relatively high creativity in dance body language and image (measured by psychometric creativity test), followed by jazz or musicals, and finally ballet dancers (Fink and Woschnjak, 2011).

To sum up, through the research of dance psychology, and the relationship between dance teaching and creativity discussed in the books of dance psychology, it is found that improvisational dance is the best training mode to cultivate dancers’ creativity. The research direction of improvisational dance and creativity is a relatively new research direction. There are not many studies on improvisational dance from the perspective of creativity, but there are many passages about improvisational dance in studies on modern dance teaching. The best way and method to discuss the improvisational dance to cultivate students’ creativity are put forward through more in-depth study and reference (Deng et al., 2021). The research conclusion of improvisational dance from the perspective of psychology is that improvisational dance is closely related to the cultivation of creativity. Generally, through the analysis of the research status in China and foreign countries, modern psychological theory is regarded as the basic concept, and its application in the cultivation of creativity in the teaching of improvisational dance is discussed.

## Materials and Methods

### Improvisational Dance Teaching Training

The relationship between improvising dance and improvisational dance can be interpreted literally. Improvising dance is a verb and improvisational dance is a noun. Improvising dance is one of the links of dance creation, and one of the topics of modern dance choreography theory, which is included in the compulsory course of the university dance department. Different from the domestic situation, foreign improvisation of dance is in the situation of diversified personality development. There is much theoretical knowledge of improvisational dance for learning, and countless improvisational dance works for learning and appreciation. Moreover, in Asia, South Korea and Japan also attach great importance to the cultivation of dancers’ creativity. They also set up the improvisational dance course as a required course in the school syllabus. The forms of improvisational dance performance can be divided into “unlimited improvisation” and “limited improvisation.” “Unlimited improvisation” is suitable for professional dancers. It can help them to be liberated from the original dance form, so that they can dance freely according to their psychological needs and body rhythm, without defining any external restrictions of theme, melody, category, and rhythm (Feng and Chen, 2020).

The choreographer or teacher gives dancers spiritual stimulation and relatively free defining space in the process of improvisation. Under this kind of stimulation, the psychological space will change greatly, and its manifestation can become extremely fragile and sensitive. Dancers can also be infinitely brave, to explore the strong demand for change and innovation; therefore, choreographers and teachers must be able to predict the role and expected results of the dancers in the body movement in the improvisational dance performance. The form of improvisational dance is that the dancer has no premeditated dance behavior and expresses the emotional expression of the dancer’s inner world in a flash through the body, which is from the inside to outside, so as to express the dancer’s current subjective consciousness. Improvisational dance can express the emotional expression of body and mind in the moment, and its form is not limited by space, time, meaning, and other factors. From the perspective of psychology, it can be understood as the coexistence of consciousness and unconsciousness, which is the representation of inner thoughts and the most creative expression. Among them, in improvisational dance, the body, as the body language of dance, is the manifestation of conscious emotion and unconscious instinctive impulse. Through the stream of consciousness and the series of different modeling changes, it reflects the emotional expression of the dancer’s heart (Fischer et al., 2019).

### Improvisational Dance and Creativity Stimulation

In psychology, there are several important schools of ideas about creativity theory. These classical ideas include Freud’s consciousness creativity and “Libido” theories, Guilford’s three-dimensional structure model of intelligence theory, Gardner’s theory of multiple intelligences, and Mihaly Csikszentmihalyi’s “flow theory.”

#### Freud’s Unconscious and Dance Creativity

Freud emphasized that psychological structure includes conscious (Cs), preconscious (PCS), and unconscious (UCS). It is believed that the psychological process is mainly unconscious. In short, unconscious and preconscious constitute their unconsciousness. Freudian psychoanalysis has three core elements: id, ego, and superego. Among them, id can be called libido, that is, unconscious (Ismail et al., 2019). This kind of id energy in the subconscious, that is, the psychological energy that stimulates behavior can be called libido, is the ability that human beings are born with. It follows the principle of happiness and meets instinctive needs. This kind of unconscious psychological activity has great significance to art creation. Freud also put forward the view on art creation, which holds that artistic creation is a process of empathy; moreover, the creation phenomenon of artworks and art was often studied through his own psychoanalytic theory, and it was believed that the unconscious thinking process is of great significance to art. Among them, the psychological process of Michelangelo’s creation of Moses was analyzed. It was believed that Moses, who breaks the traditional image of history, is a man with a sacred mission and is endowed with a new image, which reflects the artist’s inner self-reflection and criticism. Only by constantly introspecting, criticizing, and enriching the heart, can artists wake up the audience’s emotional resonance with works of art, and satisfy the audience’s unconscious desire and instinctive impulse. In improvisational dance, it can be said that many dancers’ works are completed in creative imagination through unconscious instinctive reaction (Capurso et al., 2014). To sum up, improvisational dance is closely related to Freud’s theory of unconscious creativity. Improvisational dance is a direct reflection of the dancer’s inner world of communication. Through body language expression, it can directly excavate the unconscious buried in the heart.

#### Three-Dimensional Structure Model of Intelligence Theory and Dance Creativity

In the study of creativity by the American psychologist Gilford, the various factors that affect creativity were analyzed; it was believed that human intelligence can be divided into three dimensions, and the model of “three-dimensional structure model of intelligence” was constructed (Cohen et al., 2006). In intelligence, the first dimension is content, which includes four factors: figure, symbol, semantics, and behavior; the second dimension is operation, which includes five factors: cognition, memory, divergent thinking, aggregate thinking, and evaluation; the third dimension is product, which includes six factors: unit, implication, transformation, system, relationship, and category. In the study of aptitude, it was found that many factors are related to creativity. Moreover, intelligence and creativity were distinguished (Tyagi et al., 2017). It shows that “intelligence is the ability to reprocess different information in various ways. Creativity refers to the organization of basic abilities.” For intelligence, Gilford’s intelligence operation diagram shows that intelligence is divided into memory and thinking; thinking is divided into cognition, creation, and evaluation; creation is divided into compound thinking, divergent thinking, and aggregate thinking. It suggests that in Gilford’s studies, the operation process of creativity includes the ability of divergent thinking. In-depth research on divergent thinking was carried out and it was believed that divergent thinking is the main feature of creativity. On this basis, three characteristics of divergent thinking were put forward, namely, fluency, flexibility, and uniqueness (Reiter-Palmon et al., 2019).

The essence of Gilford’s creativity theory is the three natures of divergent thinking and its uncertainty. The uncertainty of improvisational dance is closely related to modern physics and postmodern literature and art. Improvisational dance may be one of the dance forms that can best reflect divergent thinking. How to connect divergent thinking with improvisational dance is the key to the author’s thinking, because the angle of creation is multi-faceted, and all factors of improvisational dance can be reflected, such as rhythm, strength, speed, space, and so on. In improvisational dance, three ways of thinking, dance image, dance action, and dance abstract, echo each other. However, divergent and convergent thinking are mainly used to inspire the divergent thinking of college students’ dancers, so that they can feel the dance in their colorful life, open their imagination space, truly realize the unity of abstract thinking and concrete thinking in the teaching of improvisational dance, and stimulate their creativity and potential.

#### Theory of Multiple Intelligences and Dance Creativity

H. Gardner believed that multiple intelligences include nine kinds of intelligences, among which the existing intelligences are still being confirmed by research, so it can also be called eight and half intelligences. In the United States, the famous psychologist H. Gardner put forward the concept of “intelligence” in 1983. Through the continuous exploration of intelligence, nine kinds of multiple intelligences were developed and perfected (Fox, 2017), which were closely related to dance. Among them, dance has a direct relationship with body movement intelligence, music intelligence, space intelligence, and self-introspection intelligence. Dance is a dynamic plastic art with space, time, and comprehensiveness. The body is the material carrier of dance, through which dancers can express their emotions and thoughts, so dance teaching is closely related to multiple intelligences. Improvisational dance is to train dancers’ self-examination, spatial perception, dance with music, emotional communication, and body coordination, which are the key to cultivate multiple intelligences in improvisational dance (Cheedella et al., 2020). The significance of the theory of multiple intelligences in the field of dance art, therefore, is feasible.

In the process of improvisational dance, the basic process is that words (instructions) act as a trigger to stimulate the sense of innovation and produce a certain action response. This action reaction can be described as a pure stay in the response level, which is a kind of action imagination, ideas, or association. As a reaction, this association constantly becomes a new trigger point to produce innovative consciousness, and thus produce new actions (Chen, 2019).

#### Immersion Theory and Dance Creativity

Maslow’s peak experience is the root of the psychological theory of immersion experience; however, in the theory of the hierarchy of needs, the highest in the hierarchy of needs is the need for self-realization. Among them, peak experience can be understood as related to self-realization level. Peak experience can be taken as the excitement and smooth joy of dance artists in dance activities. Maslow called it peak experience, a need for self-realization (Call et al., 2018). When the improvisational dance is performed, its training purpose is to integrate the dance body from disorder to nature through the coordination of consciousness and body, the distribution of time, space, and strength. This view of integration is specially emphasized by flow theory. Therefore, these nine dimensions of flow theory coincide with the characteristics of improvisational dance. Before the beginning of dance impromptu, warm-up, changes in the environment, and the combination of warm-up, can help dancers quickly enter a state of concentration, which can be called “in the zone” in psychology (Ickes et al., 2017). According to the nine dimensions of “flow theory,” it is analyzed from the perspective of improvisation dancers.

This exploration holds that the psychological level of improvisational dance is the immersive experience, that is, one of the methods of flow theory, which can tap the inherent potential of dancers. This exploration can guide practice with theory, make people experience the state of body and mind in the improvisational dance learning, and stimulate the immersion experience from the heart.

### An Experimental Study on Improvisational Dance and Creativity

From December 2019 to March 2020, a combination of online questionnaire sampling and convenient sampling was used to select research objects. The research objects mainly included: 30 graduate students of grades 2016–2017 of XX dance college, 35 college students of the modern dance department of XX dance college, and 33 dance enthusiasts online. A total of 105 copies were collected. After screening, there were 98 valid questionnaires, and the effectivity rate was 93.33%. In the selection of research objects, the diversity of dance object types was mainly considered, so as to investigate as many audience groups as possible. The questionnaire test referred to “Cattell 16 personality factor questionnaire,” in which the research method and purpose were to measure innovation and creativity. Innovation and creativity (Pingxin Revised Edition) included six dimensions (cleverness, excitability, boldness, sensitivity, fantasy, and improvisation). According to the seven dimensions, 72 questions were selected. The experimental data were divided into two groups: the group who had learned improvisation dance and the group who had not. The results of the creativity test were analyzed to analyze the actual influence of improvisation dance on creativity.

According to statistical methods, SPSS statistical software was used for analysis. The six dimensions of the Cattell 16sp test and the items of SPSS were used as references. According to the feedback results of the questionnaire, there were 72 questions in total, and the optional dimension of each question was divided into three categories. According to the question, the answer was different. Except for a few questions, there was no distinction between “right” and “wrong” in most questions. Using SPSS software to calculate is convenient to make a more comprehensive evaluation and prediction of the experimental results, and the calculation method of independent sample *t*-test is used for multi-directional data analysis and comparison.

## Results and Discussion

### Data Analysis Results

Through the independent sample *t*-test, the differences of seven personality factors, including cleverness, excitability, boldness, sensitivity, fantasy, improvisation, and innovation creativity. Table 1 shows the results.

**Table 1:** Results of independent sample *t*-test.

Factor name	Mean (standard deviation)		*t*	*p*
	Learned improvisational dance	Have never learned improvisational dance		
Cleverness	25.32 (4.47)	25.01 (5.21)	0.391	0.693
Excitability	29.13 (4.24)	27.36 (5.39)	2.352	0.033^∗^
Boldness	29.73 (5.36)	26.75 (5.97)	3.523	0.003^∗∗^
Sensitivity	18.82 (2.12)	16.39 (2.46)	3.005	0.001^∗∗^
Fantasy	28.79 (4.28)	25.63 (5.74)	2.536	0.025^∗^
Improvisation	11.42 (1.68)	8.94 (1.85)	5.485	0.000^∗∗^
Innovation creativity	19.43 (3.28)	17.16 (2.86)	3.024	0.000^∗∗^

∗ *p* < 0.05;

∗∗ *p* < 0.01.

Table 1 shows that whether the sample has learned improvisational dance has no significant effect on cleverness (*p* > 0.05). It means that for cleverness, whether the sample has learned improvisational dance or not, all of them show consistency, and there is no difference. In addition, whether the sample has learned improvisational dance has significant effects on excitability, boldness, sensitivity, fantasy, improvisation, and innovation creativity (*p* < 0.05), which means that the samples learned and not learned improvisational dance show differences in excitability, boldness, sensitivity, fantasy, improvisation, and innovation creativity.

In this practice, the data of subjects who have learned improvisational dance and those who have not learned improvisational dance measured by Cattell’s 16 personality tests are compared. As shown in Table 2, the analysis shows that for the data of boldness, excitability, sensitivity, fantasy, improvisation, and innovation creativity, the data of students who have learned improvisational dance is higher than that of students who have not learned the improvisational dance. Improvisation and innovation creativity are higher than expected, but there is no significant difference in cleverness.

**Table 2:** Results of factor score comparison analysis.

Factor name	Type		*t*	*p*	
	Learned improvisational dance	Have never learned improvisational dance			
Cleverness	4.253	4.634	−0.723	>0.05	Not significant
Excitability	5.636	5.023	2.623	<0.05	Significant
Boldness	5.783	5.325	1.592	<0.05	Significant
Sensitivity	5.470	5.232	1.235	<0.05	Significant
Fantasy	5.470	5.232	1.302	<0.05	Significant
Improvisation	5.537	5.139	2.045	<0.05	Significant
Innovation creativity	6.256	6.193	2.832	<0.05	Significant

### Data Analysis and Discussion

The comparison results suggest that there is no significant difference in the cleverness factor. According to the conditions of the research object, its cleverness can be understood as intelligence, and genetic factors account for the majority. Although the cleverness of those who have learned improvisational dance is lower than that of those who have not. The result of innovation creativity shows that the innovative creative ability of those who have learned improvisational dance is relatively high. It indicates that there is no direct relationship between the cleverness and the learning of improvisation dance course and other personality can be improved. There is a significant difference in the boldness factor. The results show that the dancers who have learned improvisational dance dare to try new things and take risks. Many of the dancers who have not touched improvisational dance are afraid of improvisation and are not confident. For improvisational dance, daring to try and explore new things can promote the creative ability of dancers. There are significant differences in sensitivity factors. It is found that the dancers who have not learned improvisational dance often deal with things with an objective and independent attitude, which is extremely realistic. On the contrary, dancers who have learned improvisational dance prefer fantasy and art. There is a significant difference in the fantasy factor. It is found that the dancers who have not learned the improvisational dance will first consider the realistic conditions, and then make a choice, paying too much attention to the reality. Dancers who have learned improvisational dance prefer fantasy, high sensitivity to art, high excitement for things, and more imagination in improvisational dance. The significant value of the improvisation factor proves that improvisational dance has a direct relationship with the cultivation of improvisation. The experimental results show that there is a significant difference in the innovation creativity factor. Dancers who have never learned improvisational dance have relatively high innovation creativity. In addition, improvisational dance course practice can cultivate students’ ability to explore new things, enrich the dancer’s imagination, enhance the dancer’s self-recognition ability and improvisation, and finally achieve the cultivation of creativity (Wu and Wu, 2017; Wu et al., 2020).

### Suggestions and Conclusion

In the process of improvisational dance, the individual subjective experience is closely related to the breakthrough ability, which is also a method to determine whether the dancer enters the flow state. The body is the material carrier of dance, and emotional expression is the feedback of real life. Improvisational dance brings superior experience. Only with the dancer’s ability of improvisation and the integration of the body and mind, can the flow be produced, so that the state of flow can enhance the motivation of participation and help improve the dancer’s self-realization. The whole-heartedness can help the dancers to improvise. The two complement each other. When the dancers are selfless, the state of improvisational dance is the best.

This exploration holds that the purpose of improvisational dance training is to integrate the dance body from disorder to nature through the coordination of consciousness and body, the distribution of time, space, and strength. This integration view is particularly emphasized by flow. These nine dimensions coincide with the characteristics of improvisational dance. First, in the teaching process of improvisational dance, the teacher can guide the students to balance their own dance skills and the difficulty of objective things. Flow occurs when the goal challenge is balanced with one’s own ability. In the process of teaching, students as the main body need a high degree of concentration, clear internal motivation, and physical and mental unity, focusing on the current psychological state. Both teachers and students need to use the flow theory flexibly to strengthen students’ perception of the art of improvisational dance and stimulate their creativity (Yuan and Wu, 2020).

At the beginning of the improvisational dance teaching, students’ skills have not been improved, so it is difficult to achieve a high-level flow state. At this time, students need to exercise their own improvisational dance skills, and they will be slack after mastering the form of improvisational dance. Then, as the lead, the teacher should strengthen the requirements of the students, increase the difficulty of the challenge, make the students achieve the balance of challenge and skills. In improvisational dance, when the dancers reach a balance between skills and challenge goals, they will have a flow state. If the difficulty is lower than their own skills, it will produce boredom. If the difficulty is greater than their own skills, they will feel anxious. When skills and challenge difficulty balance, dancers can produce flow. The basis of its balance is not rational measurement, but subjective perceptual measurement.

## Conclusion

In improvisational dance, dancers not only mobilize innovative thinking, but also train the activity of action thinking. Under the action of creative thinking, dance works can produce unlimited possibilities and find a breakthrough for the cultivation of dance creativity, which is the most effective way to cultivate creativity. Through the analysis of the relevant theories of improvisational dance and the connection with psychological theory, the theoretical basis for training and improving creative ability of improvisation dance is found. These theoretical bases include the unconscious and libido concept of Freud’s psychoanalysis, three-dimensional structure model of intelligence theory, and the divergent thinking concept proposed by the American psychologist Gilford, the father of creative research, the immersion experience proposed by the active psychology Mihaly Csikszentmihalyi, and the multiple intelligences of psychologist Gardner. In the later stage, the creative ability of samples learned and not learned improvisational dance is analyzed by using Cattell’s 16 personality tests. The feasibility and effectiveness of the views proposed are verified by comparing the results. This exploration proves that improvisational dance is necessary for the creativity training of dancers. Improvisational dance is the art of practice, and the creative theory is the motive force of improvisational dance. Based on the analysis of the combination of theory and practice, and psychological theoretical methods, new theoretical and data support is proposed for the cultivation of college students’ dance choreography innovation.

## Data Availability Statement

The raw data supporting the conclusions of this article will be made available by the authors, without undue reservation.

## Ethics Statement

The studies involving human participants were reviewed and approved by Zhengzhou University Ethics Committee. The patients/participants provided their written informed consent to participate in this study. Written informed consent was obtained from the individual(s) for the publication of any potentially identifiable images or data included in this article.

## Author Contributions

Dancing has always been my passion. In the pursuit of dance, art and choreography, I would like to send my sincerest gratitude to my parents, partners, teachers and classmates for their support and encouragement. Professor LJ has guide me along the way and made a substantial, direct and intellectual contribution to the work. JG has been my partner and mentor since TED translation program. And I appreciate for his selfless, constructive dedication.

### Conflict of Interest

The authors declare that the research was conducted in the absence of any commercial or financial relationships that could be construed as a potential conflict of interest.

## References

[ref1] Abecassis-Moedas C. Gilson L. L. (2017). Drivers and levels of creative leadership: an examination of choreographers as directive and integrative leaders. Innovation 20, 122–138. 10.1080/14479338.2017.1394793

[ref2] Bläsing B. Calvo-Merino B. Cross E. S. Jola C. Honisch J. Stevens C. J. (2012). Neurocognitive control in dance perception and performance. Acta Psychol. 139, 300–308. 10.1016/j.actpsy.2011.12.005, PMID: 22305351

[ref3] Call A. Domenech Rodríguez M. M. Vázquez A. L. Corralejo S. M. (2018). Predicting participation in dual language immersion using theory of planned behavior. Biling. Res. J. 41, 23–36. 10.1080/15235882.2018.1425935

[ref4] Capurso V. Fabbro F. Crescentini C. (2014). Mindful creativity: the influence of mindfulness meditation on creative thinking. Front. Psychol. 4:1020. 10.3389/fpsyg.2013.01020, PMID: 24454303 PMC3887545

[ref5] Cheedella N. Andanapalli K. Cheepurupalli R. (2020). Automatic controlled wheel chair using multi-intelligence technique. J. Comput. Theor. Nanosci. 17, 2273–2278. 10.1166/jctn.2020.8883

[ref6] Chen M. (2019). The impact of expatriates’ cross-cultural adjustment on work stress and job involvement in the high-tech industry. Front. Psychol. 10:2228. 10.3389/fpsyg.2019.02228, PMID: 31649581 PMC6794360

[ref7] Chung K. H. Hou J. H. (2018). Mapping indeterminacy and chance through movement notation: a study on dance improvisation. Leonardo 51, 282–283. 10.1162/leon_a_01570

[ref8] Cohen A. Fiorello C. A. Farley F. H. (2006). The cylindrical structure of the Wechsler intelligence scale for children—IV: a retest of the Guttman model of intelligence. Intelligence 34, 587–591. 10.1016/j.intell.2006.05.003

[ref10] Deng X. Guo X. Wu Y. J. Chen M. (2021). Perceived environmental dynamism promotes entrepreneurial team member’s innovation: explanations based on the uncertainty reduction theory. Int. J. Environ. Res. Public Health 18:2033. 10.3390/ijerph18042033, PMID: 33669732 PMC7921965

[ref9] De Oliveira Moreira J. Drawin C. R. (2015). Possible relation between psychosis and the unconscious: a review of “The Unconscious,” by Freud. Front. Psychol. 6:1001. 10.3389/fpsyg.2015.01001, PMID: 26236270 PMC4502339

[ref11] Felice M. C. Alaoui S. F. Mackay W. E. (2016). “How Do Choreographers Craft Dance?” in *Proceedings of the 3rd International Symposium on Movement and Computing – MOCO’16; July 5-6, 2016.*

[ref12] Feng B. Chen M. (2020). The impact of entrepreneurial passion on psychology and behavior of entrepreneurs. Front. Psychol. 11:1733. 10.3389/fpsyg.2020.01733, PMID: 32793066 PMC7385187

[ref13] Fink A. Woschnjak S. (2011). Creativity and personality in professional dancers. Pers. Individ. Differ. 51, 754–758. 10.1016/j.paid.2011.06.024

[ref14] Fischer C. Malycha C. P. Schafmann E. (2019). The influence of intrinsic motivation and synergistic extrinsic motivators on creativity and innovation. Front. Psychol. 10:137. 10.3389/fpsyg.2019.00137, PMID: 30778313 PMC6369195

[ref15] Fisher M. Kuhlmann N. Moulin H. Sack J. Lazuk T. Gold I. (2020). Effects of improvisational dance movement therapy on balance and cognition in Parkinson’s disease. Phys. Occup. Ther. Geriatr. 38, 385–399. 10.1080/02703181.2020.1765943

[ref16] Fox S. (2017). Beyond AI: multi-intelligence (MI) combining natural and artificial intelligences in hybrid beings and systems. Technologies 5:38. 10.3390/technologies5030038

[ref17] Gillam T. (2013). Creativity and mental health care. Ment. Health Pract. 16, 24–30. 10.7748/mhp2013.06.16.9.24.e807

[ref18] Giordano G. (2000). Gaetano Grossatesta, an eighteenth-century Italian choreographer and impresario, part one: the dancer-choreographer in Northern Italy. Dance Chron. 23, 1–28. 10.1080/01472520008569369

[ref19] Haller C. S. Courvoisier D. S. (2010). Personality and thinking style in different creative domains. Psychol. Aesthet. Creat. Arts 4, 149–160. 10.1037/a0017084

[ref20] H’Doubler M. N. (1957). Dance: A Creative Art Experience. Madison, WI: University of Wisconsin Press.

[ref21] Henfield M. S. Washington A. R. Owens D. (2010). To be or not to be gifted: the choice for a new generation. Gift. Child Today 33, 17–25. 10.1177/107621751003300207

[ref22] Ickes L. Welti A. Lohmann U. (2017). Classical nucleation theory of immersion freezing: sensitivity of contact angle schemes to thermodynamic and kinetic parameters. Atmos. Chem. Phys. 17, 1713–1739. 10.5194/acp-17-1713-2017 25627933

[ref23] Ismail M. Yusof N. Rani A. (2019). Subconscious mind: a perspective from AQIDAH, SHARICA, and TASAWWUF. Humanit. Soc. Sci. Rev. 7, 555–558. 10.18510/hssr.2019.7475

[ref24] Kaufman J. C. (2005). The door that leads Into madness: eastern European poets and mental illness. Creat. Res. J. 17, 99–103. 10.1207/s15326934crj1701_8

[ref25] Kirsh D. (2014). The importance of chance and interactivity in creativity. Pragmat. Cogn. 22, 5–26. 10.1075/pc.22.1.01kir

[ref26] Kirsh D. Muntanyola D. Jao R. J. Lew A. Sugihara M. (2000). “Choreographic methods for creating novel, high quality dance.” in *Proceedings of the 5th International Workshop on Design and Semantics of Form and Movement (DeSForM)*; July 5-6, 2016; 188–195.

[ref27] Lauronen E. Veijola J. Isohanni I. Jones P. B. Nieminen P. Isohanni M. (2004). Links between creativity and mental disorder. Psychiatry 67, 81–98. 10.1521/psyc.67.1.81.31245, PMID: 15139587

[ref28] Long R. E. (2006). Broadway, the golden years: Jerome Robbins and the great choreographer-directors: 1940 to the present. London: Continuum.

[ref29] Łucznik K. (2015). Between minds and bodies: some insights about creativity from dance improvisation. Technoetic Arts 13, 301–308. 10.1386/tear.13.3.301_1

[ref30] Ludwig A. M. (1989). Reflections on creativity and madness. Am. J. Orthopsychiatry 43, 4–14. 10.1176/appi.psychotherapy.1989.43.1.4 2648869

[ref31] Megill B. (2018). New identities new voices: introducing the choreographer-notator. J. Mov. Arts Lit. 4.

[ref32] Nahrstedt K. Bajcsy R. Wymore L. Sheppard R. M. Mezur K. (2008). “Computational Model of Human Creativity in Dance Choreography.” in *AAAI Spring Symposium: Creative Intelligent Systems*; July 5-6, 2016; 53–60.

[ref33] Powell J. S. (1995). Pierre Beauchamps, choreographer to Moliere’s troupe Du Roy. Music. Lett. 76, 168–186. 10.1093/ml/76.2.168

[ref34] Reiter-Palmon R. Forthmann B. Barbot B. (2019). Scoring divergent thinking tests: a review and systematic framework. Psychol. Aesthet. Creat. Arts 13:144. 10.1037/aca0000227

[ref35] Triana D. D. (2015). The ability of choreography creative thinking on dance performance. Harmonia J. Arts Res. Educ. 15, 119–125. 10.15294/harmonia.v15i2.4555

[ref36] Tyagi V. Hanoch Y. Hall S. D. Runco M. Denham S. L. (2017). The risky side of creativity: domain specific risk taking in creative individuals. Front. Psychol. 8:145. 10.3389/fpsyg.2017.00145, PMID: 28217103 PMC5289983

[ref37] Wu W. Wang H. Wu Y. (2020). Internal and external networks, and incubatees’ performance in dynamic environments: entrepreneurial learning’s mediating effect. J. Technol. Transf. 10.1007/s10961-020-09790-w

[ref38] Wu Y. Wu T. (2017). A decade of entrepreneurship education in the Asia Pacific for future directions in theory and practice. Manag. Decis. 55, 1333–1350. 10.1108/MD-05-2017-0518

[ref39] Yuan C.-H. Wu Y. J. (2020). Mobile instant messaging or face-to-face? Group interactions in cooperative simulations. Comput. Hum. Behav. 113:106508. 10.1016/j.chb.2020.106508

